# Redox regulation of NADP-malate dehydrogenase is vital for land plants under fluctuating light environment

**DOI:** 10.1073/pnas.2016903118

**Published:** 2021-02-02

**Authors:** Yuichi Yokochi, Keisuke Yoshida, Florian Hahn, Atsuko Miyagi, Ken-ichi Wakabayashi, Maki Kawai-Yamada, Andreas P. M. Weber, Toru Hisabori

**Affiliations:** ^a^Laboratory of Chemistry and Life Science, Institute of Innovative Research, Tokyo Institute of Technology, 226-8503 Yokohama, Japan;; ^b^School of Life Science and Technology, Tokyo Institute of Technology, 226-8503 Yokohama, Japan;; ^c^Institute of Plant Biochemistry, Cluster of Excellence on Plant Sciences, Center for Synthetic Life Sciences, Heinrich Heine University Düsseldorf, 40225 Düsseldorf, Germany;; ^d^Department of Biochemistry and Molecular Biology, Graduate School of Science and Engineering, Saitama University, 338-8570 Saitama, Japan

**Keywords:** NADP-malate dehydrogenase, thioredoxin, genome editing, redox regulation, chloroplast

## Abstract

Many enzymes involved in photosynthesis possess highly conserved cysteine residues that serve as redox switches in chloroplasts. These redox switches function to activate or deactivate enzymes during light-dark transitions and have the function of fine-tuning their activities according to the intensity of light. Accordingly, many studies on chloroplast redox regulation have been conducted under the hypothesis that “fine regulation of the activities of these enzymes is crucial for efficient photosynthesis.” However, the impact of the regulatory system on plant metabolism is still unclear. To test this hypothesis, we here studied the impact of the ablation of a redox switch in chloroplast NADP-malate dehydrogenase (MDH). By genome editing, we generated a mutant plant whose MDH lacks one of its redox switches and is active even in dark conditions. Although NADPH consumption by MDH in the dark is expected to be harmful to plant growth, the mutant line did not show any phenotypic differences under standard long-day conditions. In contrast, the mutant line showed severe growth retardation under short-day or fluctuating light conditions. These results indicate that thiol-switch redox regulation of MDH activity is crucial for maintaining NADPH homeostasis in chloroplasts under these conditions.

## Significance

Plant chloroplasts have acquired an evolutionary “redox switch” that regulates the activities of photosynthesis-related enzymes in response to a dynamically changing environment. Because the metabolic state in chloroplasts drastically fluctuates in response to changes in the surrounding environment, it has been considered essential to adjust the activities of chloroplast enzymes. Many efforts have been made to elucidate this regulation in detail. Its physiological role is thought to be due to the fact that fine-tuning of the activity is essential for efficient photosynthesis, but it is still unclear how this is really the case. We have shown that the redox switch of NADP-malate dehydrogenase plays a crucial role in the optimal growth of plants under fluctuating light conditions and prolonged darkness.

The thiol-based redox regulation system plays a critical role in the regulation of photosynthesis. Many photosynthetic enzymes possess conserved cysteine pairs, which serve as redox switches (1, 2). These enzymes are usually activated by the reduction of cysteines under photosynthetic conditions and are deactivated by oxidation in the dark (3). This reductive activation system, which is mediated by a ubiquitous redox-responsive protein thioredoxin (Trx), was discovered in the 1970s (4). In contrast, the oxidation process in chloroplasts was recently suggested to be mediated by Trx and Trx-like proteins (5–8). They receive reducing equivalents from the redox-regulated proteins and transfer electrons to H_2_O_2_ via 2-Cys peroxiredoxins. This redox-based photosynthesis regulation system is considered to be one of the most important strategies for the acclimation of sessile plants to fluctuating light environment (9, 10).

Redox switches are conserved in many photosynthesis-related enzymes, such as the four Calvin–Benson cycle enzymes glyceraldehyde-3-phosphate dehydrogenase, fructose-1,6-bisphosphatase (FBPase), sedoheptulose-1,7-bisphosphatase, and phosphoribulokinase (1, 9). Further, thylakoid ATP synthase and the NADP-malate dehydrogenase (MDH) are redox-regulated (1, 9). MDH works in the malate valve and produces malate using NADPH to export the reducing power from chloroplast to the cytosol. This valve system is considered to be important to balance the ATP/NADPH ratio in chloroplasts (11). Since nonplastidial-type homologs of these enzymes do not possess the redox switch and are constitutively active without reduction by Trx, it is assumed that the redox switch serves to suppress the enzymatic activity particularly in dark conditions. There are several hypotheses as to why dark deactivation is needed: FBPase, to prevent futile ATP consumption with the reaction catalyzed by phosphofructokinase (9); ATP synthase, to prevent a futile reverse reaction, ATP hydrolysis (12, 13); MDH, to prevent the depletion of reducing power in chloroplasts caused by excessive export of the reducing power via the malate valve (14–16). In particular, the activity of MDH in the dark is strictly regulated by two switches at the N terminus and C terminus of the enzyme molecule, suggesting that the complete deactivation of MDH must be critical as one of the acclimation responses (14, 16). In addition, fine regulation of the activities of chloroplast enzymes such as FBPase and MDH in response to light intensity and metabolic state in chloroplasts is very important for the metabolic homeostasis (3, 17). Studies on the redox regulation of chloroplast enzymes were potentially motivated by these hypotheses, although the impact of the redox regulation system on plant metabolism is still unclear. Therefore, it is important to clarify the physiological significance of redox regulation, particularly pertaining to the deactivation in the dark and fine-tuning in the light. To this end, we developed mutant plants in which the thiol-based redox switch of the strictly regulated enzyme MDH was deleted in the native genomic context by clustered regularly interspaced short palindromic repeat (CRISPR)/CRISPR-associated nuclease 9 (Cas9)-mediated genome editing. Redox regulation of MDH appears dispensable under standard long-day conditions. Instead, we demonstrated that the redox regulation of MDH is essential in the extended dark periods and under the fluctuating light conditions.

## Results

### Biochemical Characterization of Chloroplast MDH without a C-Terminal Redox Switch.

**Fig. 1.**
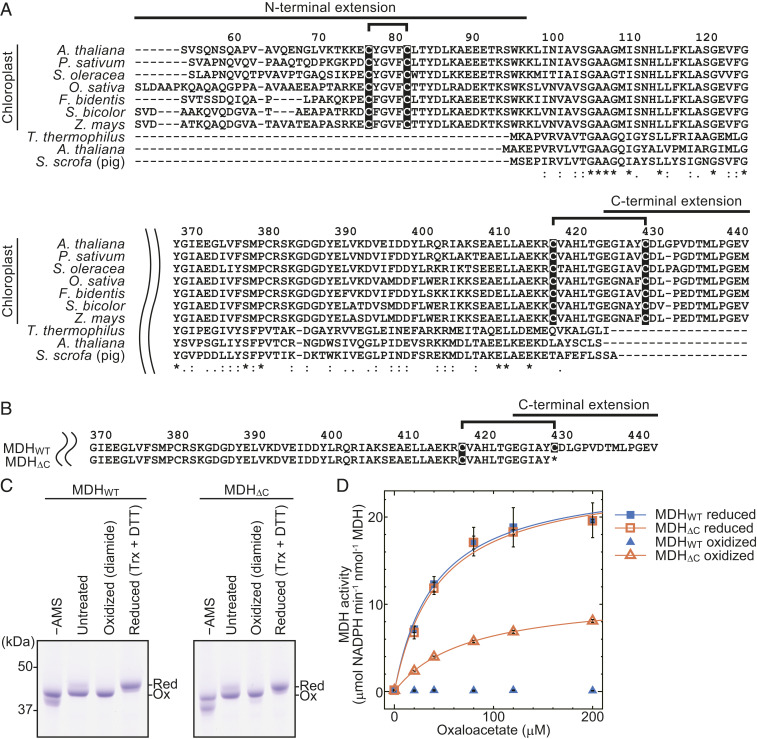
Redox-regulated cysteine residues in MDH and their impact on the MDH activity. (*A*) Amino acid sequence comparison of N- and C-terminal extension regions of MDHs. Amino acid sequences are available from the UniProtKB database with accession nos. (chloroplast MDH from *A. thaliana*, Q8H1E2; *Pisum sativum*, P21528; *Spinacia. oleracea*, P52426; *Oryza* *sativa*, Q6YYW3; *Flaveria* *bidentis*, P46489; *Sorghum* *bicolor*, P17606; and *Zea* *mays*, P15719; and nonchloroplast MDH from *Thermus* *thermophilus*, P10584; *A. thaliana*, P93819; and *Sus* *scrofa*, P11708). Residues highlighted in black are redox-regulated cysteines. The residue numbers of chloroplast MDH from *A. thaliana* (counted from the translational start site methionine) are shown above the sequence. The consensus symbols (*, fully conserved; :, strongly conserved; and ., weakly conserved) obtained via the Clustal Omega analysis (https://www.ebi.ac.uk/Tools/msa/clustalo/) are shown below the alignment result. (*B*) The amino acid sequence of the C-terminal region of the MDH_ΔC_ mutant. Residues highlighted in black are redox-regulated cysteines. The residue numbers of chloroplast MDH from *A. thaliana* (counted from the translational start site methionine) are shown above the sequence. (*C*) Redox state of recombinant MDH used for measuring activity. MDH_WT_ and MDH_ΔC_ were treated with oxidant diamide or reductant DTT and Trx. After the treatment, reduced (Red) and oxidized (Ox) MDH proteins were discriminated via thiol group modification using 4-acetamido-4′-maleimidylstilbene-2,2′-disulfonate followed by sodium dodecyl sulfate-polyacrylamide gel electrophoresis (SDS-PAGE). SDS-PAGE was performed using 10% acrylamide gel containing 6 M urea. (*D*) Activities of recombinant MDH_WT_ and MDH_ΔC_. Values are presented as mean ± SD (*n* = 3, one measurement for each independently prepared (pretreated) sample) and fitted to the Michaelis–Menten equation. The fitting curve for oxidized MDH_WT_ could not be obtained due to almost zero *V*_max_.

The regulatory mechanism of chloroplast MDH in terms of its biochemical and structural aspects has been well studied (9, 14, 18–33). In contrast to cytosolic MDH, MDH has two cysteine pairs, which serve as redox switches, in the N- and C-terminal amino acid extensions of the protein (Fig. 1*A*). Based on the crystal structure of the molecule and results of previous biochemical studies, the formation of a disulfide bond on the C-terminal extension may induce conformational changes, which place the C terminus in close proximity to the active site, consequently preventing substrate binding (14, 20–25, 27–30). Hence, this C-terminal extension is thought to be critical for the deactivation of MDH (14, 28, 29). We therefore tested whether ablation of the C-terminal switch leads to a constitutively active enzyme by using a recombinant MDH whose redox switch-containing C-terminal extension was deleted. Note that only the region from the second cysteine of the C-terminal switch (Fig. 1*B*, cysteine 430) to C terminus was deleted, because the first cysteine is located in the core region of the MDH molecule (Fig. 1*A*, cysteine 418) and deletion from the position of the first cysteine resulted in insoluble proteins (27). Hereinafter, we designated the mutant MDH_ΔC_ and studied its substrate usage efficiency. Activities of recombinant MDH proteins with and without the C-terminal extension (WT [MDH_WT_] and MDH_ΔC_) were then compared. When they were reduced by Trx (Fig. 1*C*), both proteins displayed almost the same activities (Fig. 1*D*, filled and open squares, respectively). When oxidized, MDH_WT_ became completely deactivated, in agreement with several previous studies (22, 26, 27, 33), whereas MDH_ΔC_ retained 30–45% of its maximal activity (Fig. 1*D*, filled and open triangles, respectively). These results indicate that MDH_ΔC_ is constitutively active, although the N-terminal redox switch apparently controls approximately half of the activity.

### Generation of a Mutant Plant Expressing MDH_ΔC_.

**Fig. 2.**
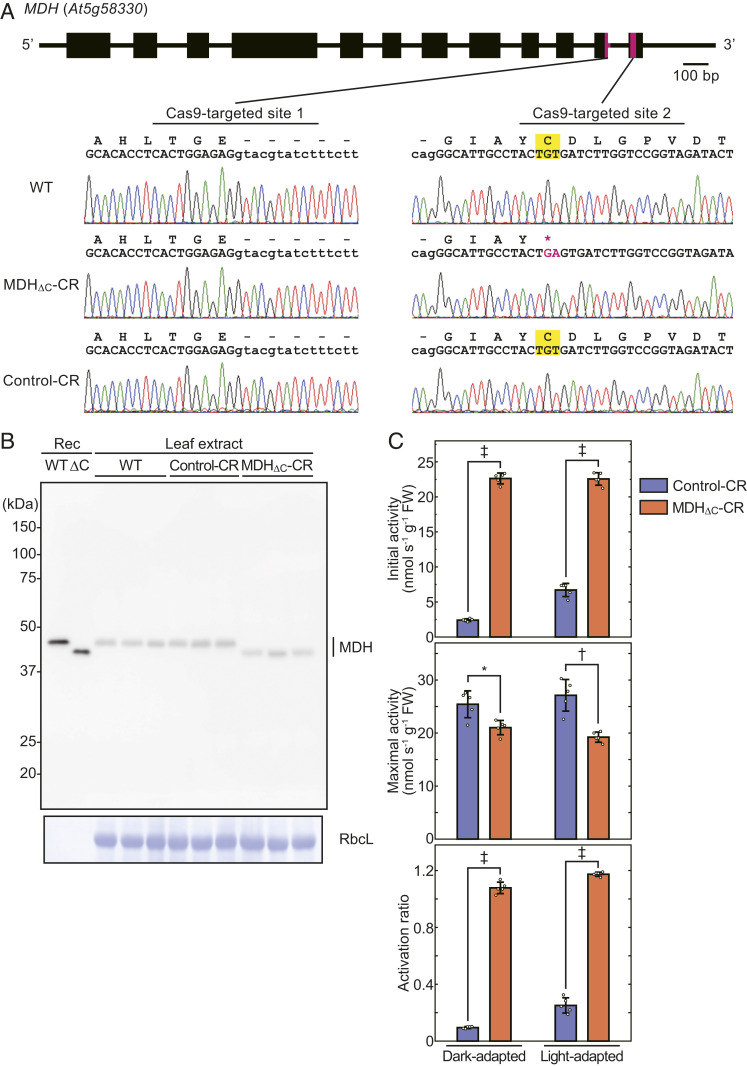
Confirmation of the introduction of the ΔC mutation into the *Arabidopsis* genome. (*A*) Mutated site in *MDH* gene. Cas9-targeted sites are shown as magenta bars in *MDH* gene structure. DNA sequencing results for each strain are shown with translated amino acid sequences. The mutated site is shown in magenta in the DNA sequencing result and amino acid sequence. Letters highlighted in yellow represent the redox-regulated cysteines and codons that code the cysteines. (*B*) The expression of MDH proteins in plant leaves. Rosette leave extract of each strain (three biological replicates) was subjected to SDS-PAGE followed by Western blot analysis with anti-MDH antibodies. Recombinant (Rec) MDH proteins were used as markers. CBB-stained Rubisco large subunit (RbcL) is shown as a loading control. (*C*) The activation ratio of MDH in rosette leaf extract. MDH was extracted from dark-adapted (8 h) or light-adapted (60 μmol photons m^−2^·s^−1^, 16 h) plant leaves and subjected to activity measurement. The activation ratio was determined using values of initial and maximal activities. Each value is presented as mean ± SD (*n* = 5, biological replicates). Each symbol indicates significant difference (*P < 0.05; †*P* < 0.01; ‡*P* < 0.001; Welch’s *t* test) between the values of Control-CR and MDH_ΔC_-CR plants.

Next, we introduced the MDH_ΔC_ mutation into the gene in the *Arabidopsis* genome using the CRISPR/Cas9-mediated genome editing system, which can introduce point mutations, such as insertions or deletions, onto a target site (34–36). To delete the regulatory C-terminal domain of the protein, we aimed to introduce a stop codon prior to this domain. We then chose two target sites in the chloroplast MDH gene (At5g58330) as shown in Fig. 2*A*. The vector design used for plant transformation is described in *SI Appendix*, Fig. S1. G and A nucleotides were introduced into the TGT codon portion encoding the second cysteine. As a result, the sequence TGAGT was generated, and a stop codon TGA appeared, which caused a deletion of the C-terminal extension similar to that of the recombinant MDH_ΔC_ (Fig. 2*A*). We therefore named this mutant plant line as MDH_ΔC_-CR (“CR” comes from CRISPR/Cas9). We also obtained a plant line that was transformed with the same CRISPR/Cas9 construct but harbored no mutation in the *MDH* gene (Fig. 2 *A*, *Lower*). This line (Control-CR) was used as a nonedited negative control. Note that both MDH_ΔC_-CR and Control-CR lines no longer possess a transfer DNA (T-DNA) insertion including a Cas9-expression cassette in and after T2 generations.

We then confirmed the CRISPR/Cas9-induced mutation at both the RNA and protein levels. As shown in *SI Appendix*, Fig. S2, the introduction of the desired ΔC mutation was observed in messenger RNA (mRNA). Western blot analysis showed that the apparent molecular mass of MDH proteins in MDH_ΔC_-CR plants was smaller than that in WT and Control-CR plants, similar to the recombinant proteins (Fig. 2*B*). It was also evident that the MDH expression levels were not remarkably altered in MDH_ΔC_-CR plants (Fig. 2*B*). The enzymatic activity of MDH in leaf extract (initial activity) was also measured and compared to the activity of a fully reduced enzyme (maximal activity) (Fig. 2*C*) (37). MDH proteins in MDH_ΔC_-CR plants were fully active under both dark and light conditions, and the activity was significantly higher than that in Control-CR plants (Fig. 2*C*). However, MDH proteins in both lines in the dark, which should be oxidized (3), showed higher activities than expected from the experiments with recombinant proteins (Fig. 1*D*), and the activation ratio in MDH_ΔC_-CR plants was greater than 1 (Fig. 2*C*). These phenomena were probably due to the effect of dithiothreitol (DTT), because 5 mM DTT was used to suppress oxidation for initial activity measurement, and 100 mM DTT was used for maximal activity measurement according to the conventional method (37). Indeed, high concentrations of DTT can inhibit the activities of some enzymes in a redox-independent manner (38). Nevertheless, we could confirm that MDH in MDH_ΔC_-CR plants certainly functioned in vivo, and its regulation system, namely the deactivation in the dark and fine-tuning in the light, was compromised. These results indicate that the genome-edited MDH_ΔC_-CR line is a useful model for the examination of the role of the C-terminal redox switch of MDH in vivo.

### Physiological Significance of the Redox Regulation of MDH.

**Fig. 3.**
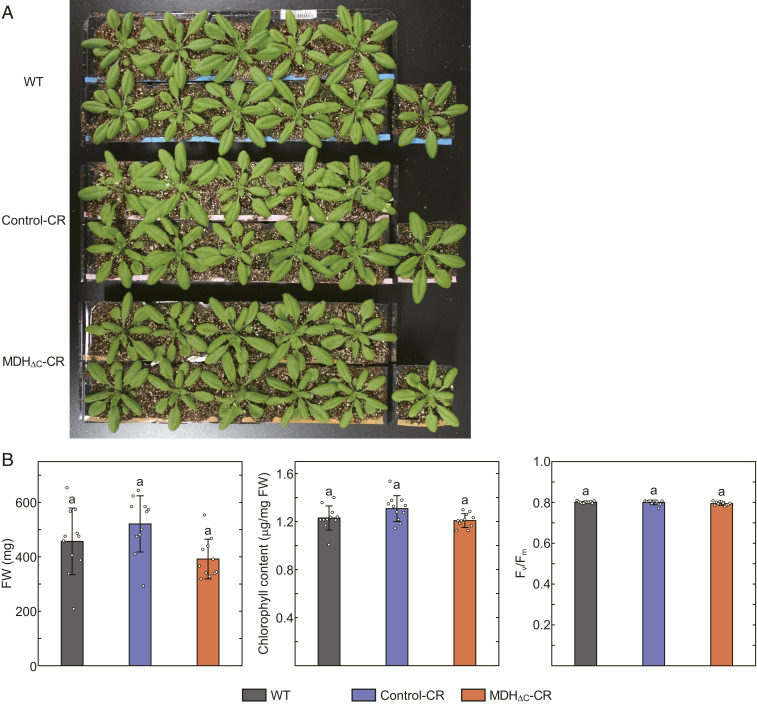
Phenotypes of plants grown under LD-GL conditions. (*A*) Plants grown under LD-GL conditions for 28 d. (*B*) Physiological parameters of plants grown under LD-GL conditions. Each value is presented as mean ± SD (*n* = 11, biological replicates). Different letters indicate significant differences among plants (*P* < 0.01, one-way ANOVA and Tukey honest significance difference [HSD]).

We investigated the physiological significance of the redox regulation of MDH by using the MDH_ΔC_-CR plant. We first grew plants under long-day (LD) (16-h light/8-h dark) conditions at a growth light (GL) intensity of 60 μmol photons m^−2^·s^−1^. We designated these LD-GL conditions as normal growth conditions. Under these conditions, the visible phenotype (Fig. 3*A*) and physiological parameters such as fresh weight (FW), chlorophyll content, and maximum quantum yield of photosystem II (F_v_/F_m_) (Fig. 3*B*) were indistinguishable from control lines. It was previously assumed that MDH activity must be strictly suppressed in dark conditions. However, our results indicate that MDH activity in the dark is not detrimental, at least under conditions with an 8-h dark period per day.

**Fig. 4.**
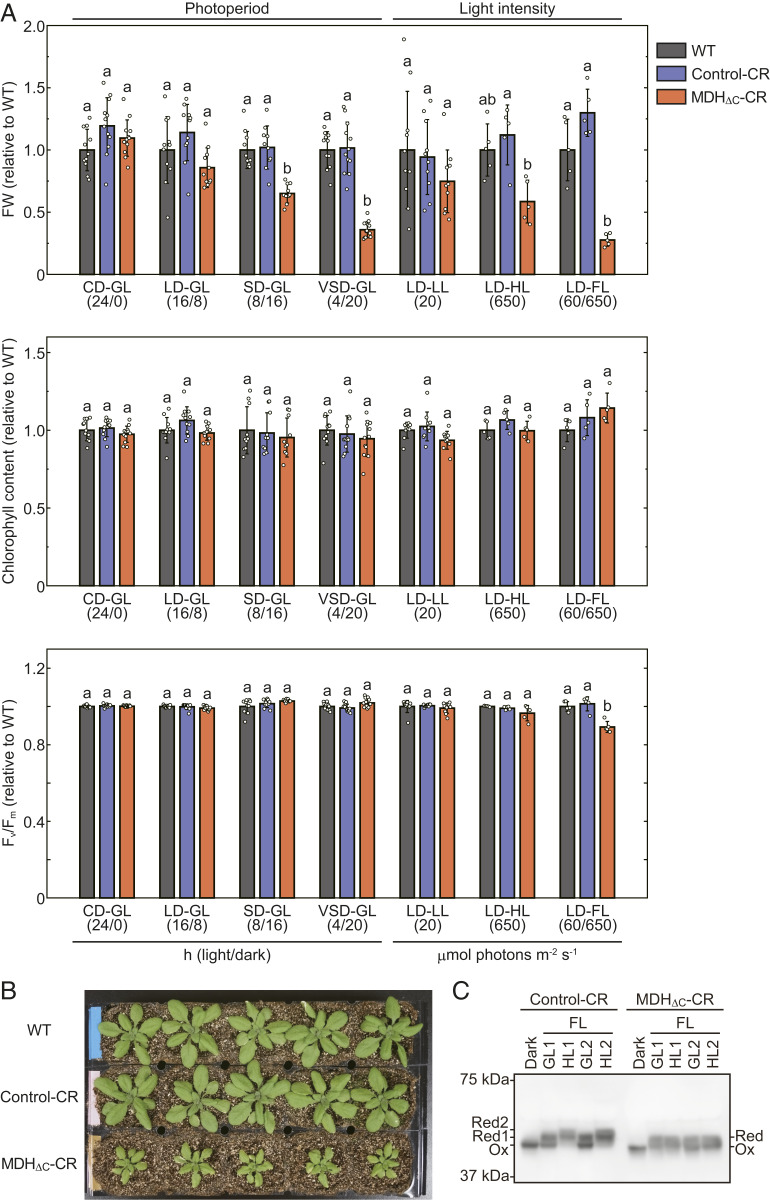
Phenotypes of plants grown under various conditions. (*A*) Physiological parameters of plants grown under various conditions. The data shown in Fig. 3*B* were used for calculation regarding LD-GL conditions. Plants were grown for 26 d (CD-GL), 42 d (SD-GL), 56 d (VSD-GL), 34 d (LD-LL), 22 d (LD-HL), or 23 d (LD-FL). Light/dark term (hours) or light intensity (μmol photons m^−2^·s^−1^) is described in parentheses below conditions. Each value was normalized by using the value of WT grown under the same conditions and presented as mean ± SD (*n* ≥ 5, biological replicates). Different letters indicate significant differences among plants grown under the same conditions (*P* < 0.01, one-way ANOVA and Tukey HSD). (*B*) Plants grown under LD-FL conditions for 23 d. (*C*) Redox state of MDH under FL conditions. Plants were grown under LD-GL conditions for 28 d and dark-adapted for 8 h. Subsequently, these plants were put under LD-FL conditions. Using plant leaves before illumination (Dark), just before the end of the first GL and HL term (GL1 and HL1), and just before the end of the second GL and HL term (GL2 and HL2), the redox state of MDH was determined essentially according to a previous report (66). Reduced (Red) and oxidized (Ox) MDH proteins were discriminated via thiol group modification using 4-acetamido-4′-maleimidylstilbene-2,2′-disulfonate followed by SDS-PAGE and Western blot analysis.

Although the C-terminal redox switch of MDH does not seem to be essential under LD-GL conditions, it is thought to play a critical role when plants are subjected to certain growth conditions because the redox switches of chloroplast enzymes, including MDH, are highly conserved among land plants. Thus, we next examined the effect of the ΔC mutation on plant growth under various conditions in terms of variations in the length of the light period. We examined continuous-day (CD) (24-h light/0-h dark), short-day (SD) (8-h light/16-h dark), and very short-day (VSD) (4-h light/20-h dark) conditions at GL intensity. As shown in Fig. 4*A* (Photoperiod lanes), the FW of MDH_ΔC_-CR plants relative to WT plants gradually decreased depending on the length of the dark period. In contrast, chlorophyll content and F_v_/F_m_ of MDH_ΔC_-CR plants were comparable to those of WT and Control-CR plants. These results indicate that the deactivation of MDH in the dark is advantageous for plant growth during the light/dark cycle, particularly under conditions with long dark periods, but this deactivation is not critical for maintaining the photosynthetic apparatus in chloroplasts.

As previously reported, the activities of most of the chloroplast redox-regulated enzymes are fine-tuned, depending on light intensity and metabolic state in chloroplasts (3, 11, 17, 39, 40). Therefore, another possible role of redox regulation is suggested to be maintaining the redox balance in chloroplasts under conditions with varying or fluctuating light intensity. Hence, we applied LD conditions with different light intensities such as low light (LL) (20 μmol photons m^−2^·s^−1^) and high light (HL) (650 μmol photons m^−2^·s^−1^) instead of GL. In addition, we used fluctuating light (FL) conditions, where the light intensity changes every 30 min between GL and HL. We found that MDH_ΔC_-CR plants showed severe growth retardation and decreased F_v_/F_m_ values under LD-FL conditions, and these effects were more drastic than those that were observed under VSD-GL conditions (Fig. 4 *A* and *B*). Similar results were obtained using another line expressing MDH without the C-terminal extension (*SI Appendix*, Figs. S2 and S3). In contrast to this, the phenotypes of MDH_ΔC_-CR plants grown under LD-LL and LD-HL conditions were less pronounced when compared with plants under LD-FL conditions (Fig. 4*A*, Light intensity lanes; Fig. 4*B*; and *SI Appendix*, Fig. S4). In addition, we obtained an MDH-deficient T-DNA insertion line (*nadp-mdh*, Salk_012655C) from the *Arabidopsis* Biological Resource Center. The line obtained from the same parental line (Salk_012655) was characterized in a previous study (15). We used it to produce mutant plants overexpressing MDH_WT_, MDH_ΔC_, or another mutant MDH whose four cysteines of both N- and C-terminal redox switches were replaced by serine (MDH_CtoS_) (MDH_WT_-OE, MDH_ΔC_-OE, and MDH_CtoS_-OE, respectively; *SI Appendix*, Fig. S5). Mutant plants expressing MDH_ΔC_ or MDH_CtoS_ presented similar phenotypes as MDH_ΔC_-CR plants under LD-FL conditions, supporting the importance of the redox switch under LD-FL conditions, whereas they exhibited retarded growth even under LD-GL conditions (*SI Appendix*, Fig. S5).

We also examined the redox state of MDH under FL conditions. MDH in both Control-CR and MDH_ΔC_-CR plants was fully oxidized in the dark (Fig. 4*C*). According to the results from our biochemical study, MDH in MDH_ΔC_-CR plant in the dark should have about half of the maximal activity, although it showed almost full activity in Fig. 2*C* probably due to the effect of DTT. The redox state of MDH in the Control-CR plant dramatically changed under FL conditions (Fig. 4*C*), indicating that the MDH activity is regulated in response to changes in light intensity, whereas the redox state of MDH in MDH_ΔC_-CR plant was constant. These results suggest that the MDH redox switch is particularly important for plant growth under FL environments.

### Metabolite Dynamics in the Mutant Plant.

**Fig. 5.**
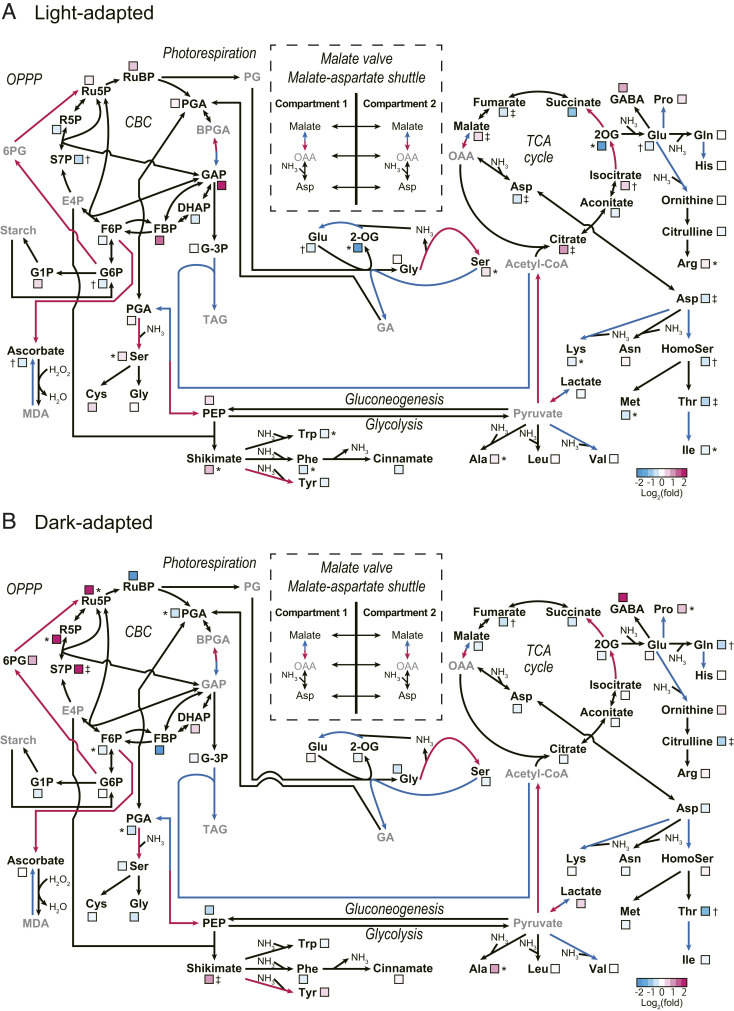
Schematic metabolite maps of MDH_ΔC_-CR plants. (*A*) Metabolite alterations in light-adapted (16 h) MDH_ΔC_-CR plants compared with those in Control-CR plants. (*B*) Metabolite alterations in dark-adapted (8 h) MDH_ΔC_-CR plants compared with those in Control-CR plants. (*A* and *B*) Plants used for metabolome analysis were grown under LD-GL conditions for 4 wk. The ratio of each metabolite content in MDH_ΔC_-CR and Control-CR plants was normalized by Log_2_(fold) and was displayed as a color box based on the metabolite data shown in *SI Appendix*, Table S1 (*n* = 5, biological replicates). Cyan and magenta arrows represent NADPH consuming and producing reactions, respectively. Metabolites shown in gray were not determined or not detected. Each symbol indicates significant difference (*P < 0.05; †*P* < 0.01; ‡*P* < 0.001; Welch’s *t* test) between the values of Control-CR and MDH_ΔC_-CR plants. A map for the malate valve and malate-aspartate shuttle is shown without metabolite level data. The metabolome data shown here indicate overall levels of metabolites in leaf extracts. 2OG, 2-oxoglutarate; 6PG, 6-phosphogluconate; BPGA, 1,3-bisphosphoglycerate; CBC, Calvin–Benson cycle; DHAP, dihydroxyacetone phosphate; E4P, erythrose-4-phosphate; F6P, fructose-6-phosphate; FBP, fructose-1,6-bisphosphate; G1P, glucose-1-phosphate; G-3P, glycerol-3-phosphate; G6P, glucose-6-phosphate; GA, glycerate; GABA, γ-aminobutyrate GAP, glyceraldehyde-3-phosphate; HomoSer, homoserine; MDA, monodehydroascorbate; OAA, oxaloacetate; OPPP, oxidative pentose-phosphate pathway; PEP, phosphoenolpyruvate; PG, 2-phosphoglycolate; PGA, 3-phosphoglycerate; R5P, ribose-5-phosphate; Ru5P, ribulose-5-phosphate; RuBP, ribulose-1,5-bisphosphate; S7P, sedoheptulose-7-phosphate; TAG, triacylglycerol; TCA, tricarboxylic acid.

MDH_ΔC_-CR plants did not exhibit retarded growth under LD-GL conditions (Fig. 3), while MDH in MDH_ΔC_-CR plants might be active even in the dark (Figs. 1*D* and 2*C*). To clarify why MDH activity in the dark did not affect the plant growth under LD-GL conditions, we performed a metabolome analysis of mutant plants under light and dark conditions. The results are summarized in *SI Appendix*, Table S1, and the metabolic maps are shown in Fig. 5 to easily find the relationships among metabolites. The raw metabolome data shown in *SI Appendix*, Table S1 are overall levels of metabolites in leaf extracts. In light-adapted MDH_ΔC_-CR plant, the malate content was significantly higher than that in the Control-CR plant (Fig. 5*A*). In contrast, a remarkable decrease in aspartate (Asp) and amino acids derived from Asp, such as threonine (Thr), was observed (Fig. 5*A*). These results imply that the malate valve and malate-Asp shuttle are accelerated, and/or the amount of oxaloacetate for the Asp aminotransferase reaction is low, probably due to a highly active mutant MDH in chloroplasts. If the activated malate valve exports reducing power from chloroplasts to the cytosol and other organelles, the reducing power, namely NAD(P)H, should be increased in these other compartments. As shown in Fig. 5*A*, contents of citrate and isocitrate, which are substrates of the NADH production steps during the tricarboxylic acid (TCA) cycle, were increased, while the products of these steps, 2-oxoglutarate (2OG), succinate, and fumarate, were decreased. Although the results shown in Fig. 5*A* do not indicate the metabolite contents only in mitochondria, they may reflect the inhibited NADH production steps during the TCA cycle in MDH_ΔC_-CR plants, probably due to feedback inhibition caused by the increased NADH level (41). In dark-adapted MDH_ΔC_-CR plants, most of these differences caused by the deletion of the redox switch were insignificant, and consequently, the malate content was not significantly different from that observed in Control-CR plants (Fig. 5*B*). In contrast, the intermediate products of the oxidative pentose-phosphate pathway (OPPP), such as ribulose-5-phosphate (Ru5P), ribose-5-phosphate (R5P), and sedoheptulose-7-phosphate (S7P), increased significantly (Fig. 5*B*). These data suggest the up-regulation of OPPP, a pathway for NADPH production, in the dark, and this point needs to be thoroughly investigated in the future.

**Fig. 6.**
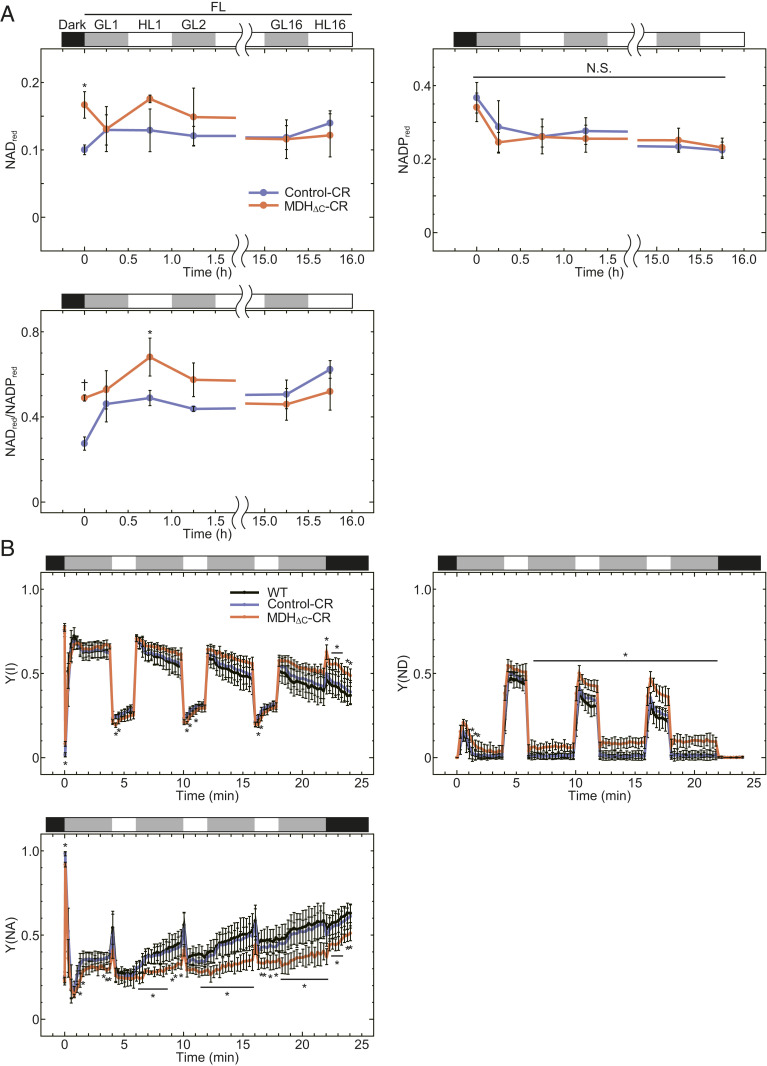
Redox state of NAD(P) pool under FL conditions. (*A*) A reduced fraction of NAD or NADP (NAD_red_ or NADP_red_) and the ratio of them in leaf extracts. NAD_red_ and NADP_red_ were calculated by using the data shown in *SI Appendix*, Fig. S6. Each value is presented as mean ± SD (*n* = 3, biological replicates). Each symbol indicates significant difference (*P < 0.05; †*P* < 0.01; Welch’s *t* test) between the values of Control-CR and MDH_ΔC_-CR plants. Black, gray, and white bars represent light intensities, 0, 60, and 650 μmol photons m^−2^·s^−1^. N.S., not significant. (*B*) Photosynthetic parameters related to PSI capacity under fluctuating light conditions. Each value is presented as mean ± SD (*n* = 5, biological replicates). Each symbol indicates a significant difference (*P < 0.05; one-way ANOVA and Tukey HSD) between the values of MDH_ΔC_-CR plants and both WT and Control-CR plants. Any significant differences were not detected between the values of WT and Control-CR plants. Black, gray, and white bars represent light intensities, 0, 70, and 670 μmol photons m^−2^·s^−1^. (*A* and *B*) Plants grown under LD-GL conditions for 4 wk were dark-adapted for 8 h and then used for the analyses.

To investigate the underlying cause of the severe growth retardation of the MDH_ΔC_-CR plants under FL conditions, we examined NAD(P)(H) dynamics in plants under FL conditions. As a result of NAD(P)(H) quantification using leaf extracts (*SI Appendix*, Fig. S6), the reduced fraction of NAD (NAD_red_) and the ratio of NAD_red_ to NADP_red_ were larger in MDH_ΔC_-CR plants in the dark as well as in the early stages of FL conditions (Fig. 6*A*). We then analyzed photosynthetic electron transport parameters to dissect the redox state in chloroplasts. As shown in *SI Appendix*, Fig. S7, parameters related to the photosystem II (PSII) capacity were almost the same among WT, Control-CR, and MDH_ΔC_-CR plants. In contrast, parameters related to the PSI capacity such as PSI quantum yield [Y(I)], donor-side limitation [Y(ND)], and acceptor-side limitation [Y(NA)], were substantially different (Fig. 6*B*). In particular, Y(ND) and Y(NA) of MDH_ΔC_-CR plants progressively became higher and lower, respectively, than those of both WT and Control-CR plants. Contrary to this, the low Y(NA) value observed on MDH_ΔC_-CR plant was not obvious in the continuous GL or HL compared with FL (*SI Appendix*, Fig. S8). These results indicate that the oxidized NADP pool and ferredoxin, which serve as electron acceptors for PSI, are increased in MDH_ΔC_-CR plants.

## Discussion

Instead of genome editing, the overexpression of mutant proteins by exogenous expression cassettes has been conventionally used to analyze the function of a protein domain in vivo. However, this approach may not faithfully represent the sole effect of the mutation, because the expression level of the desired protein encoded by an exogenous gene will typically be different from that by the endogenous gene due to chromatin state and other factors. Spatiotemporal expression control, such as tissue-specific and circadian-dependent expression, should also be considered. In addition, several previous studies suggest that the expression level of MDH directly influences plant growth (42, 43), although Hebbelmann et al. (15) showed that MDH deficiency does not affect the growth. Overexpression of the constitutively active MDH should have a larger effect on growth; thus, the results obtained by conventional strategies, such as that shown in *SI Appendix*, Fig. S5, might reflect the combined effects of mutations in the MDH itself and the expression levels of MDH. The results that MDH_ΔC_-OE and MDH_CtoS_-OE exhibited the retarded growth even under LD-GL conditions (*SI Appendix*, Fig. S5) might be the consequence of these effects. In addition, the effect might also be one of the reasons for previous results that could not show the beneficial effect of redox switches on plant growth (13, 44, 45).

In this study, we introduced a mutation into the endogenous *MDH* gene to directly delete its C-terminal regulatory domain using the CRISPR/Cas9-mediated genome editing technology. This system is generally used for the knockout of a target gene, and the strategy of using this system in studies such as this is still rare particularly in the case of land plants. Consequently, we were able to generate plants expressing MDH_ΔC_, which was confirmed by both RNA and protein-level analyses (*SI Appendix*, Fig. S2 and Fig. 2*B*). In addition, we observed higher activity of MDH in MDH_ΔC_-CR plant leaves (Fig. 2*C*), while the data of the enzyme activity might be somewhat influenced by DTT. The metabolome data may be affected by the higher activity of MDH and malate valve in MDH_ΔC_-CR plant (Fig. 5). Hence, we suppose that our mutant line is useful for the investigation of the in vivo function of the C-terminal region of MDH.

Since redox regulation is thought to be central to plant acclimation to dynamically changing environments, many studies, including our previous biochemical study (8), have been conducted to study this mechanism in detail. As mentioned, the important functions of this system were supposed to be “deactivation of photosynthesis in the dark” and “fine-tuning of photosynthesis in the fluctuating light.” We therefore tested this fundamental hypothesis using the newly generated mutant plant. Our data indicate that the redox regulation of MDH is important for plant growth in the dark and under FL conditions.

Contrary to our expectation, there was no significant difference between long-day–grown Control-CR plants and MDH_∆C_-CR plants when NADP(H) levels in leaves and Y(NA) values in both plants were compared in the dark (Fig. 6 and *SI Appendix*, Fig. S6). The excessive consumption of NADPH in MDH_ΔC_-CR plant chloroplasts in the dark was supposed to be compensated by an up-regulation of OPPP capacity (Fig. 5*B*). We, therefore, examined transcript amounts of glucose-6-phosphate dehydrogenase (G6PDH) (*SI Appendix*, Fig. S9), which catalyzes the first committed step of OPPP and largely contributes to the determination of the whole reaction rates of OPPP (46). The transcript amounts of cytosolic G6PDH6 and chloroplastic G6PDH2, G6PDH3, and G6PDH4 (46) were increased in MDH_ΔC_-CR plants in the dark, although the increase of the G6PDH2 transcript was not significant, G6PDH3 may not be a major chloroplast isoform, and G6PDH4 is not an active isoform (46). Because G6PDH1, a major chloroplast G6PDH, was not up-regulated, transcriptional regulation of G6PDH may not contribute to the up-regulation of OPPP. Hence, the cause of changes in metabolites related to OPPP in MDH_ΔC_-CR plants including the involvement of other factors should be investigated in the future (46–49). Both the activity and redox state of MDH itself are known to be controlled by the feedback regulation by NADP(H) (*SI Appendix*, Fig. S10) (17, 33). Although this feedback regulation might not completely suppress the activity of MDH_ΔC_, it may also contribute to suppress NADPH depletion in the dark. Note that these compensatory pathways and feedback regulation system may not be sufficient for longer periods of darkness, because MDH_ΔC_-CR plants showed significant growth retardation under SD-GL and VSD-GL conditions (Fig. 4*A*).

As shown in Fig. 4, the growth retardation caused by the ΔC mutation in MDH was most prominent under LD-FL conditions. Thus, we measured the redox state of the NAD(P) pool in leaf extracts under FL conditions to clarify the reason for this growth retardation (Fig. 6*A*). Based on the results of NAD(P)(H) quantification, we found that the NAD pool in MDH_ΔC_-CR plant leaf extracts was increasingly reduced, as compared to that in Control-CR plant leaf extracts at the beginning of the light term, implying high malate valve activity in MDH_ΔC_-CR plants. However, this difference was not observed anymore at the end of the light term, probably because the effect of the chloroplast MDH activity on the whole-cell NAD(P) pool was unremarkable. However, the redox state of chloroplast NADP pool, estimated by the PSI capacity, was significantly oxidized in MDH_ΔC_-CR plants under FL conditions (Fig. 6*B*). Chloroplast NADPH is required for various important processes, such as CO_2_ fixation, fatty acid production, chlorophyll biogenesis, and detoxification of reactive oxygen species (ROS). Because excessive light energy produces ROS, such as H_2_O_2_, which are harmful to cells, chloroplasts are equipped with efficient enzymatic systems to detoxify ROS (50, 51). Most of these enzymes, including ascorbate peroxidase, glutathione peroxidase, and 2-Cys peroxiredoxin, ultimately use NADPH as a reducing power source. It is therefore likely that the chloroplast NADP pool in the MDH_ΔC_-CR plant is more oxidized and cannot supply enough reducing power to the ROS detoxification pathways. Moreover, the malate valve is supposed to be important for intracellular redox signaling that regulates peroxisomal catalase activity and H_2_O_2_ level and, subsequently, induces stress-related gene expression (43). Hence, plants without properly regulated malate valve may be easily damaged by ROS under photooxidative stress conditions. In line with this, MDH_∆C_-CR plant showed low F_v_/F_m_ under FL conditions (Fig. 4*A*).

The reducing power for redox regulation is provided by the photosynthetic electron transport system, and the rate of the electron transport is affected by many parameters, such as temperature, humidity, and CO_2_ concentration, in addition to light intensity (52). Therefore, the redox regulation system may be needed for optimizing photosynthesis by sensing and subsequently reacting to these changing parameters. Because the growth of MDH_ΔC_-CR plant was retarded in FL, the fluctuation of other parameters can similarly affect the growth of the MDH_ΔC_-CR plant. It is known that these parameters are easily and quickly changed in natural settings (52), whereas very long complete darkness, such as under VSD conditions, is unusual. In general, plants overcome these long-term stress conditions or the defect of certain genes (e.g., deficiency of MDH) by transcriptional regulation (15, 53, 54). In contrast, for the fluctuating conditions caused by short-term environmental change, more quickly responding systems such as redox regulation must be effective. For instance, the important role of Trx-*m*, which is related to the redox regulation of MDH, in photosynthesis under FL conditions is reported (53). Thus, we concluded that the redox regulation of the chloroplast MDH is more critical under ever-fluctuating environments than in darkness.

One of the important findings in this study is that, at least when expressed in its native genomic context, the redox regulation of MDH is not critical for growth under constant growth conditions in laboratories (LD-GL conditions) (Fig. 3), which is inconsistent with the prevailing model. If the redox regulation of photosynthesis-related enzymes is not essential under nonstressful conditions, it probably limits maximum photosynthesis activity under these conditions, because it suppresses the activities of several enzymes even in the light. For example, sedoheptulose-1,7-bisphosphatase (SBPase), which catalyzes one of the rate-limiting steps of the Calvin–Benson cycle, is not fully activated even under HL conditions (3, 54). Given that the overexpression of SBPase enhances plant photosynthesis and growth (55), the deletion of the redox switch of SBPase may result in the increase of maximum SBPase activity, thus leading to increased plant growth. In fact, overexpression of cyanobacterial FBP/SBPase, which has both FBPase and SBPase activity and does not possess the redox switch, greatly enhances plant growth (56, 57). Moreover, overexpression of NADPH-Trx reductase C and cystathionine β-synthase domain-containing protein, which enhances the reductive activation of chloroplast redox-regulated enzymes, also promotes plant growth (10, 58, 59). These previous studies suggest that the deletion of redox switches of chloroplast enzymes to achieve maximum activity of these enzymes is a viable strategy for increasing plant biomass under optimal growth conditions, such as those used in agriculture under controlled conditions. To determine effective targets for improving plant growth, we need to conduct further studies to understand the role of redox regulation for each target enzyme.

## Materials and Methods

### Protein Expression and Purification.

For MDH_WT_ expression, a plasmid encoding mature *Arabidopsis* MDH (starting from serine 54) with a start codon was constructed based on a previously constructed plasmid (33), whose backbone is the pET-23c vector (Novagen). For MDH_ΔC_ expression, a site-directed mutation was introduced into the plasmid using PrimeSTAR Mutagenesis Basal Kit (Takara) and primers described in *SI Appendix*, Table S2. Proteins were expressed in *Escherichia coli* and purified according to our previous report (33) with an additional size exclusion chromatography step using a Superdex 200 10/300 GL column (GE Healthcare). Recombinant *Arabidopsis* Trx-*f*1 was prepared according to our previous study (8).

### Determination of MDH Activity.

Recombinant MDH activity was determined as described in our previous report (33). MDH (2 μM) was prereduced with 1 μM Trx-*f*1 and 0.5 mM DTT, or preoxidized with 20 μM diamide for 1 h in the buffer containing 50 mM Tris⋅HCl (pH 7.5) and 50 mM NaCl at 25 °C. MDH activity was measured by monitoring the NADPH oxidation at 340 nm in the reaction buffer containing 50 mM Tris⋅HCl (pH 7.5), 50 mM NaCl, 0.01 μM MDH, 100 μM NADPH, and 0–200 μM oxaloacetate at 25 °C. For the experiment of *SI Appendix*, Fig. S10, 0–4,000 μM NADP^+^ was also added into the reaction buffer. The specific activity of MDH was determined as the velocity of NADPH oxidation (μmol NADPH·min^−1^) per 1 μmol MDH. Activity of MDH in leaf extracts was determined according to methods used in previous studies (37, 60) using plants grown under LD-GL conditions for 4 wk. MDH activation ratio was determined with the values of initial activity and maximal activity, which were measured using 5 and 100 mM DTT, respectively.

### CRISPR/Cas9-Mediated Genome Editing.

The expression plasmid containing a *Streptococcus pyogenes*-derived Cas9 and two single guide RNAs (sgRNAs) was constructed according to Hahn et al. (61), using the pFH6_new vector, pUB-Cas9 vector, and primers shown in *SI Appendix*, Table S2. To express two sgRNAs from one construct, we simultaneously integrated two sgRNA expression cassettes into the pUB-Cas9 vector via Gibson assembly using primers FH41, FH42, FH254, and FH255. Plant transformation and screening were also performed according to Hahn et al. (61) using primers described in *SI Appendix*, Table S2. *Arabidopsis thaliana* Columbia-0 was used as a WT.

### Sequence Analysis and Quantification of mRNA.

Total RNA was extracted from frozen leaves by using TRIzol Reagent (Invitrogen) according to the manufacturer’s instructions, and complementary DNA (cDNA) was synthesized via reverse transcription with ReverTra Ace polymerase (TOYOBO) and random primers. For sequence analysis, the gene of interest was amplified by PCR with synthesized cDNA and primers described in *SI Appendix*, Table S2. For quantifying the mRNA amount of G6PDH in leaves, plants grown under LD-GL conditions for 4 wk and subsequently dark-adapted for 8 h were used. Quantitative PCR was performed using synthesized cDNA, StepOnePlus Real-Time PCR System (Applied Biosystems), and THUNDERBIRD SYBR qPCR Mix (TOYOBO), and the primers used are described in *SI Appendix*, Table S2. Each value is normalized with the value of *RPS15aA* (*At1g07770*), which encodes one of the ribosomal proteins and used for normalization in previous studies (40, 62).

### Measurement of Growth and Photosynthetic Parameters.

The plants were first grown under LD-GL conditions (16-h light/8-h dark cycle, 60 μmol photons m^−2^·s^−1^, 22 °C) for 7 d and then transferred to the experimental conditions described in each figure legend. FW was determined using plants above ground. Chlorophyll was extracted from frozen leaves with 80% (vol/vol) acetone, and chlorophyll content was determined through spectrophotometry in accordance with previous research (6). Photosynthetic parameters, such as F_v_/F_m_, Y(I), Y(NA), Y(ND), Y(II), Y(NPQ), and Y(NO), were determined by measuring the chlorophyll-*a* fluorescence and P700 state with a Dual-PAM-100 spectrometer (Walz).

### Overexpression of MDH in Plants.

For MDH overexpression (OE), a kanamycin resistance cassette of the pRI 201-AN vector (Takara) was replaced by a hygromycin resistance cassette. Four cysteine residues of the N- and C-terminal redox switches of *MDH* gene (cysteine 77, 82, 418, and 430) in the pET-23c vector were replaced by serine (CtoS) by site-directed mutagenesis. Each mature MDH (WT, ΔC, or CtoS)-coding region in the pET-23c vector was fused to the transit peptide of MDH through overlap PCR and integrated into the plant transformation vector via Gibson assembly. An MDH-deficient plant line (*nadp-mdh*, Salk_012655C) was obtained from the *Arabidopsis* Biological Resource Center and transformed with the resulting vector via the *Agrobacterium*-mediated floral dip method. Homozygous mutant lines were selected and used for the experiments. Primers used for this study are shown in *SI Appendix*, Table S2.

### Metabolome Analysis.

Plants grown under LD-GL conditions for 4 wk underwent metabolite quantification. Plants were dark-adapted for 8 h or light-adapted for 16 h prior to sampling. Metabolites were extracted and quantified with a capillary electrophoresis-triple quadrupole mass spectrometry system (Agilent) as described by Miyagi et al. (63, 64) with minor modifications. For the filtration of extracts, a 3-kDa cutoff filter (Millipore) was used. Each quantification was performed with five biological replicates.

### NAD(P)(H) Quantification.

Plants grown under LD-GL conditions for 4 wk were used for NAD(P)(H) quantification. Plants were dark-adapted for 8 h and subsequently put under a 30-min GL/30-min HL cycle. NAD(P)(H) was extracted at an indicated time and quantified by plate reader assay as described in a previous report (65) with modifications as follows: values were normalized by FW instead of chlorophyll content; absorbance at 595 nm was measured every 10 s; and reaction mixtures were shaken during intervals with a microplate reader iMark (Bio-Rad).

## Supplementary Material

Supplementary File

## Data Availability

All study data are included in the article and/or *SI Appendix*.
