# Supplementary material for: Redox regulation of NADP-malate dehydrogenase is vital for land plants under fluctuating light environment
Source: Proc Natl Acad Sci U S A. 2021 Feb 2;118(6):e2016903118. doi: 10.1073/pnas.2016903118 (PMC8017969; doi:10.1073/pnas.2016903118)
Supplement: Supplementary File [file pnas.2016903118.sapp.pdf]

PNAS
www.pnas.org

Supplementary Information for

Redox regulation of NADP-malate dehydrogenase is vital for land plants under fluctuating light environment

Yuichi Yokochia, Keisuke Yoshida, Florian Hahn, Atsuko Miyagi, Ken-ichi Wakabayashi, Maki Kawai-Yamada, Andreas P. M. Weber, Toru Hisabori

Toru Hisabori
Email: thisabor@res.titech.ac.jp

This PDF file includes:

SI appendix Figures. S1 to S10
SI appendix References
SI appendix Tables. S1 to S2

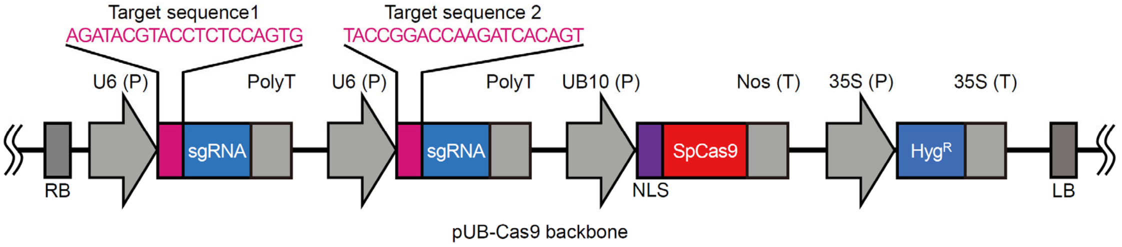

SI appendix Fig. S1. CRISPR/Cas9 construct for plant transformation. A plasmid structure between the right border (RB) and left border (LB) is shown with target sequences in single guide RNA (sgRNA). The pUB-Cas9 vector[1] was used as a backbone. Abbreviations: U6 (P), *U6-26* promoter; PolyT, poly thymidine transcription terminator; UB10 (P), *UBIQUITIN10* promoter; NLS, nuclear localization signal; SpCas9, *Streptococcus pyogenes*-derived Cas9; Nos (T), nopaline synthase terminator; 35S (P), *Cauliflower Mosaic Virus* 35S promoter; Hyg^R, hygromycin resistance gene cassette; 35S (T), *Cauliflower Mosaic Virus* 35S terminator.

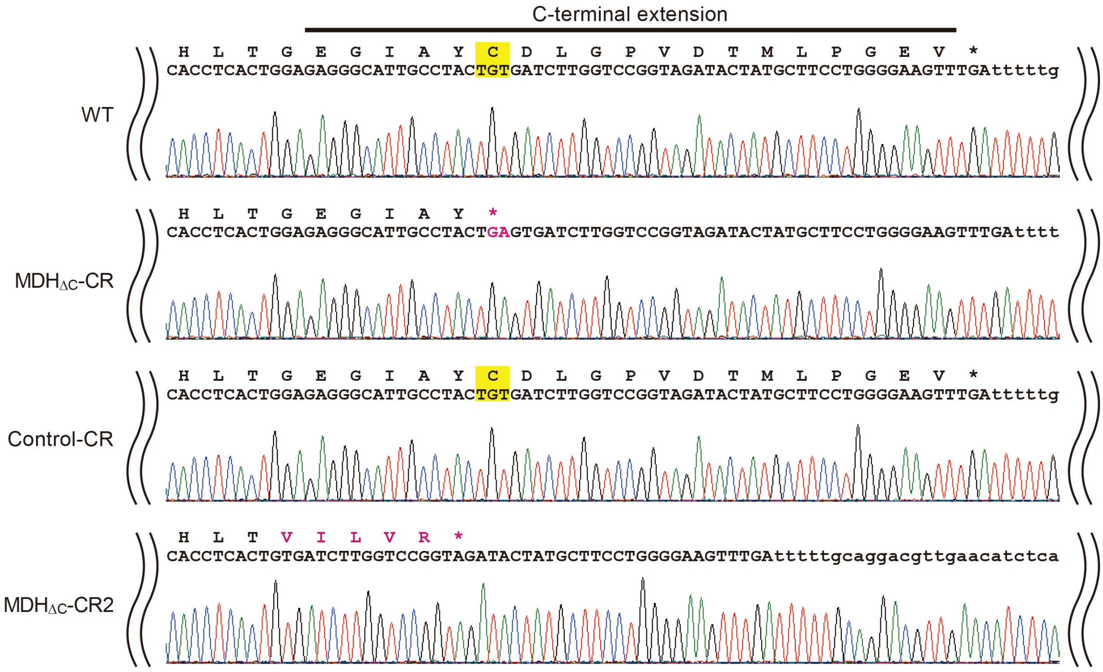

SI appendix Fig. S2. mRNA sequences of MDH. cDNA was synthesized from the RNA extract of each plant strain and its sequencing result is shown along with a translated amino acid sequence. MDH$_{\Delta C}$-CR2 is another line that expresses MDH without the C-terminal extension. The mutated sites are shown in magenta in the DNA and amino acid sequences. Letters highlighted in yellow represent the redox-regulated cysteines and codons that code the cysteines.

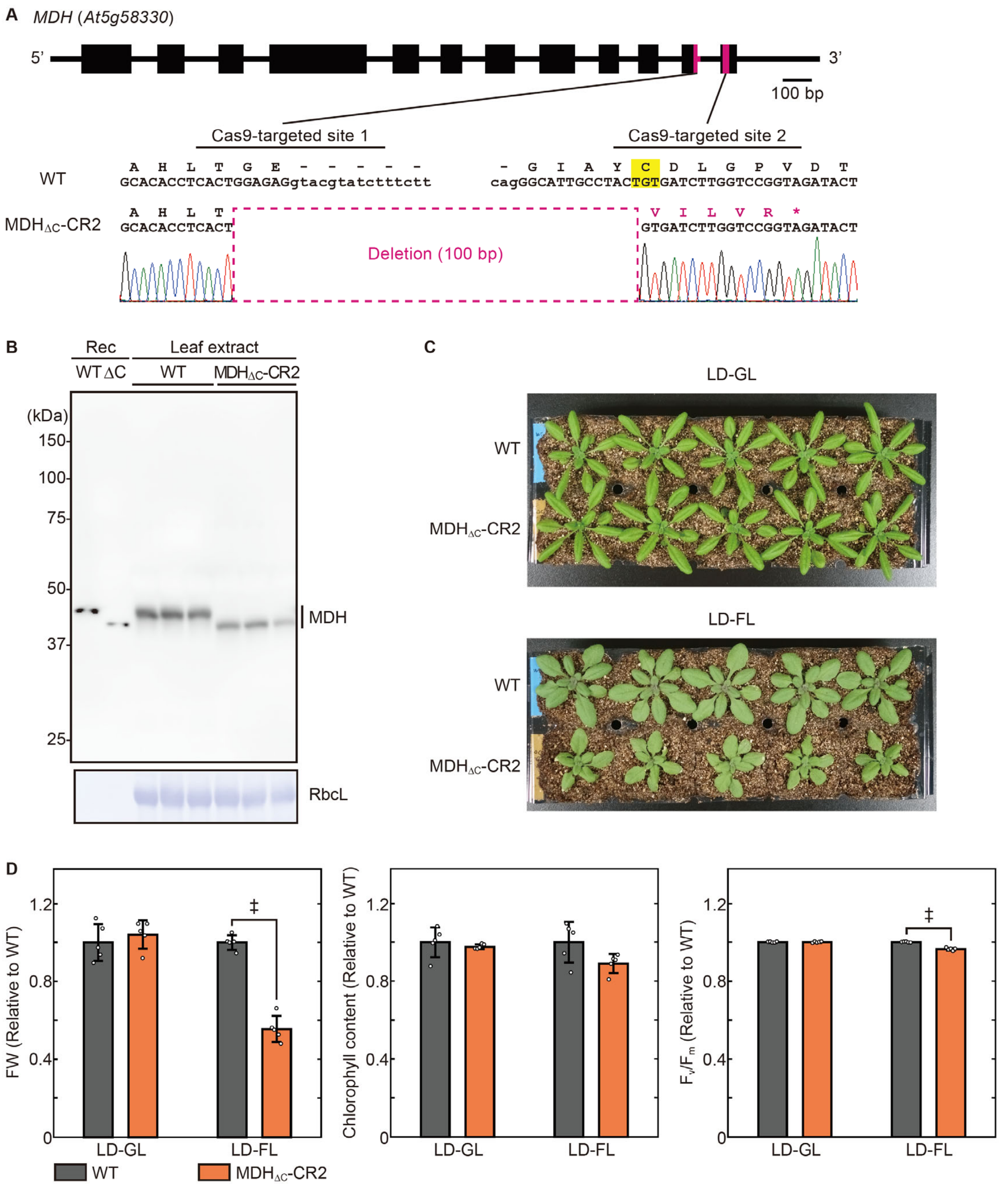

SI appendix Fig. S3. Physiological analyses of another line expressing MDH without the C-terminal extension ($MDH_{\Delta C}$-CR2). (A) Mutated site in *MDH* gene. Cas9-targeted sites are shown as magenta bars in *MDH* gene structure. A DNA sequencing result for each strain is shown with a translated amino acid sequence. The deleted DNA region is shown in magenta in the DNA sequencing result, and altered amino acids are also shown in magenta. Letters highlighted in yellow represent the redox-regulated cysteine and codon that codes the cysteine. (B) The

expression of MDH proteins in plant leaves. Rosette leave extract of each strain (3 biological replicates) was subjected to SDS-PAGE followed by Western blot analysis with anti-MDH antibodies. Recombinant (Rec) MDH proteins were used as markers. CBB-stained Rubisco large subunit (RbcL) is shown as a loading control. (C) Plants grown under LD-GL conditions for 28 days or LD-FL conditions for 23 days. (D) Physiological parameters of plants grown under LD-GL or LD-FL conditions. Each value is normalized by using the value of WT grown under the same conditions and presented as mean ± SD (n = 5, biological replicates). Each symbol indicates a significant difference (‡, $P < 0.001$; Welch's *t*-test) between the values of WT and $MDH_{\Delta C}$-CR2 plants.

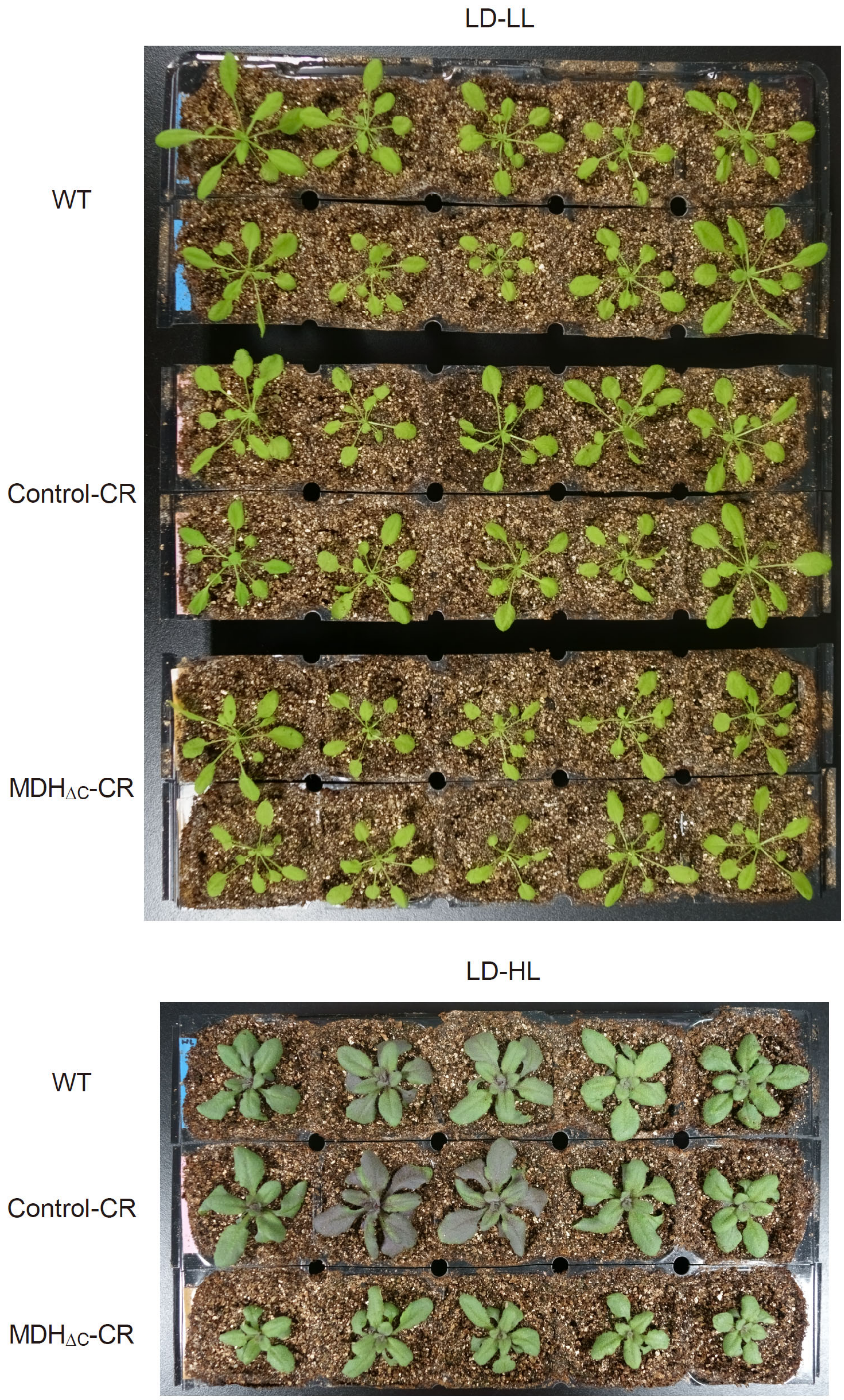

SI appendix Fig. S4. Plants grown under conditions with high or low light intensity. Plants were grown under the indicated conditions for 34 days (LD-LL) or 22 days (LD-HL).

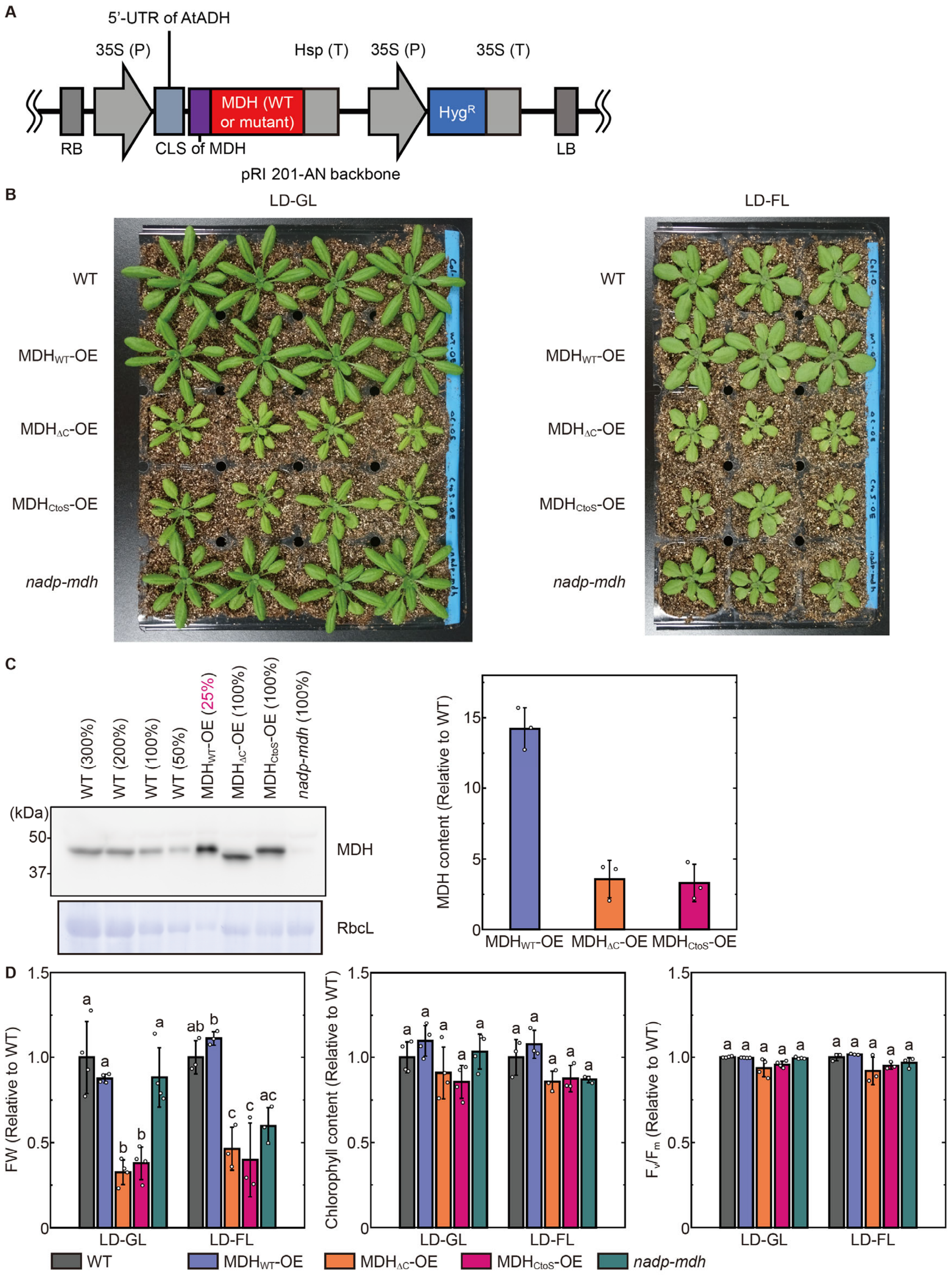

SI appendix Fig. S5. Physiological analyses of plants overexpressing WT or mutant MDH. (A) The construct for plant transformation and MDH overexpression (OE). A plasmid structure between the right border (RB) and left border (LB) is shown. The pRI 201-AN vector was used

as a backbone. Abbreviations: 35S (P), Cauliflower Mosaic Virus 35S promoter; AtADH, *A. thaliana* alcohol dehydrogenase; CLS, chloroplast localization signal; Hsp (T), heat shock protein terminator; Hyg^R, hygromycin resistance gene cassette; 35S (T), Cauliflower Mosaic Virus 35S terminator. (B) Plants grown under LD-GL conditions for 28 days or LD-FL conditions for 23 days. *nadp-mdh* is an MDH-deficient plant line, and this was used as a background for plant transformation. (C) MDH content in each mutant plant. Values in parentheses represent a relative loaded protein amount. CBB-stained Rubisco large subunit (RbcL) is shown as a loading control. MDH content determined by measuring band intensity is presented as mean ± SD (n = 3, biological replicates). (D) Physiological parameters of plants grown under LD-GL or LD-FL conditions. Each value is normalized by using the value of WT grown under the same conditions and presented as mean ± SD (n = 4 (LD-GL) or 3 (LD-FL), biological replicates). Different letters indicate significant differences among plants grown under the same conditions ($P < 0.01$, one-way ANOVA and Tukey HSD).

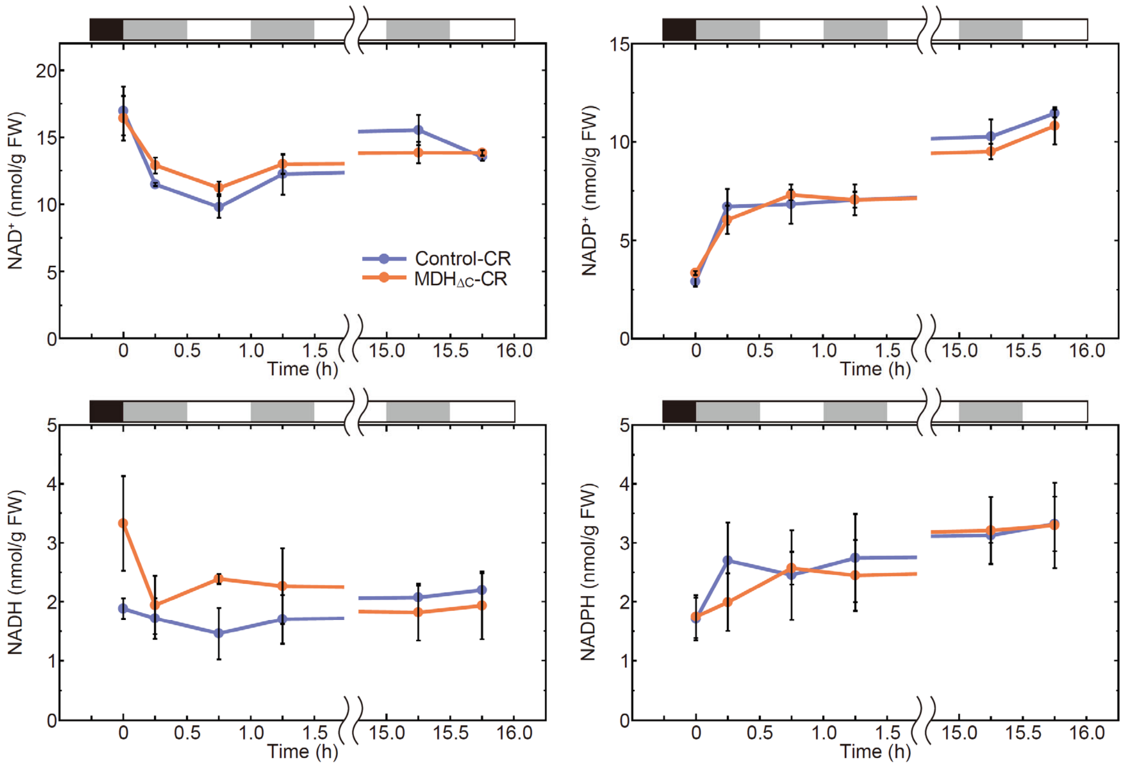

SI appendix Fig. S6. NAD(P)(H) dynamics in leaf extracts. Each value is presented as the mean ± SD (n = 3, biological replicates). Black, gray, and white bars represent light intensities, 0, 60, and 650 µmol photons m^{-2} s^{-1}. Plants grown under LD-GL conditions for 4 weeks were dark-adapted for 8 h and then used for the analyses.

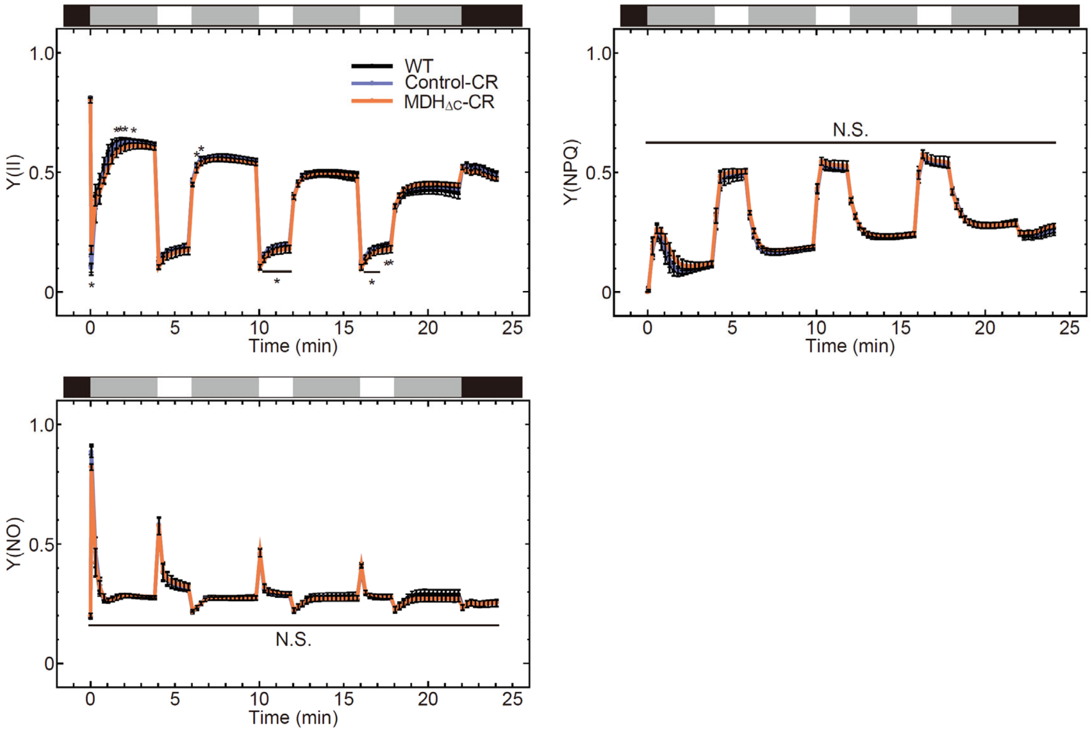

SI appendix Fig. S7. Photosynthetic parameters related to PSII capacity under fluctuating light conditions. Each value is presented as mean ± SD (n = 5, biological replicates). Each symbol indicates a significant difference (*, $P < 0.05$; one-way ANOVA and Tukey HSD) between the values of MDH$_{\Delta C}$-CR plants and both WT and Control-CR plants. Any significant differences were not detected between the values of WT and Control-CR plants. In any point of Y(NPQ) and Y(NO), differences of values among plants were not significant (N.S.). Black, gray, and white bars represent light intensities, 0, 70, and 670 μmol photons m^{-2} s^{-1}. Plants grown under LD-GL conditions for 4 weeks were dark-adapted for 8 h and then used for the analyses.

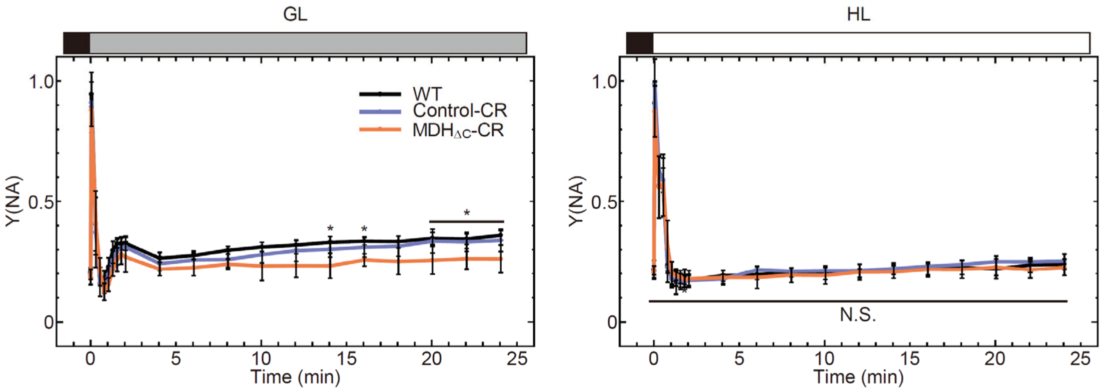

SI appendix Fig. S8. Transition of Y(NA) value under continuous GL or HL conditions. Each value is presented as mean ± SD (n = 5, biological replicates). Each symbol indicates a significant difference (*, P < 0.05; one-way ANOVA and Tukey HSD) between the values of MDH$_{\Delta C}$-CR plants and both WT and Control-CR plants. Any significant differences were not detected between the values of WT and Control-CR plants. Under HL conditions, differences of values among plants were not significant (N.S.). Black, gray, and white bars represent light intensities, 0, 70, and 670 μmol photons m^{-2} s^{-1}. Plants grown under LD-GL conditions for 4 weeks were dark-adapted for 8 h and then used for the analyses.

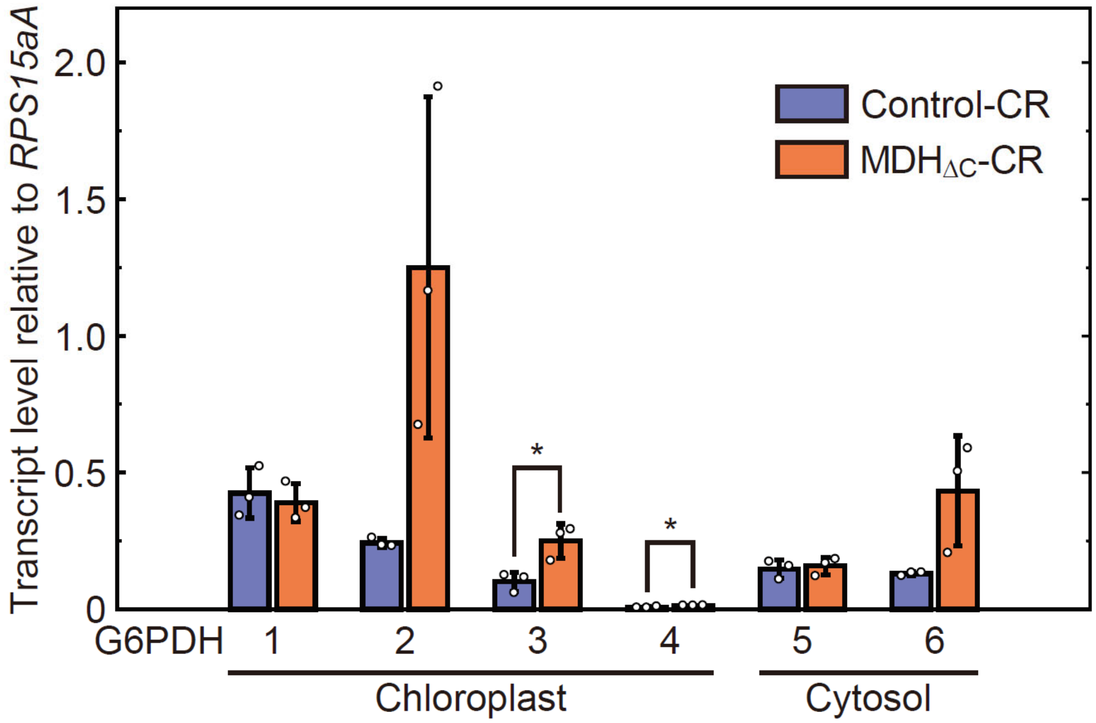

SI appendix Fig. S9. Transcript levels of G6PDH isoforms in the dark. Transcript levels of *G6PDH1* (*At5g35790*), *G6PDH2* (*At5g13110*), *G6PDH3* (*At1g24280*), *G6PDH4 (At1g09420*), *G6PDH5* (*At3g27300*), *G6PDH6* (*At5g40760*), and *RPS15aA* (*At1g07770*) genes were determined by quantitative PCR. G6PDH isoforms 1–4 are localized in chloroplasts, and isoforms 5 and 6 are localized in the cytosol[2]. Each value is normalized by the transcript level of *RPS15aA* gene (*At1g07770*), which encodes one of the ribosomal proteins, and presented as mean ± SD (n = 3, biological replicates). Each symbol indicates a significant difference (*, $P < 0.05$; Welch's *t*-test) between the values of Control-CR and $MDH_{\Delta C}$-CR plants.

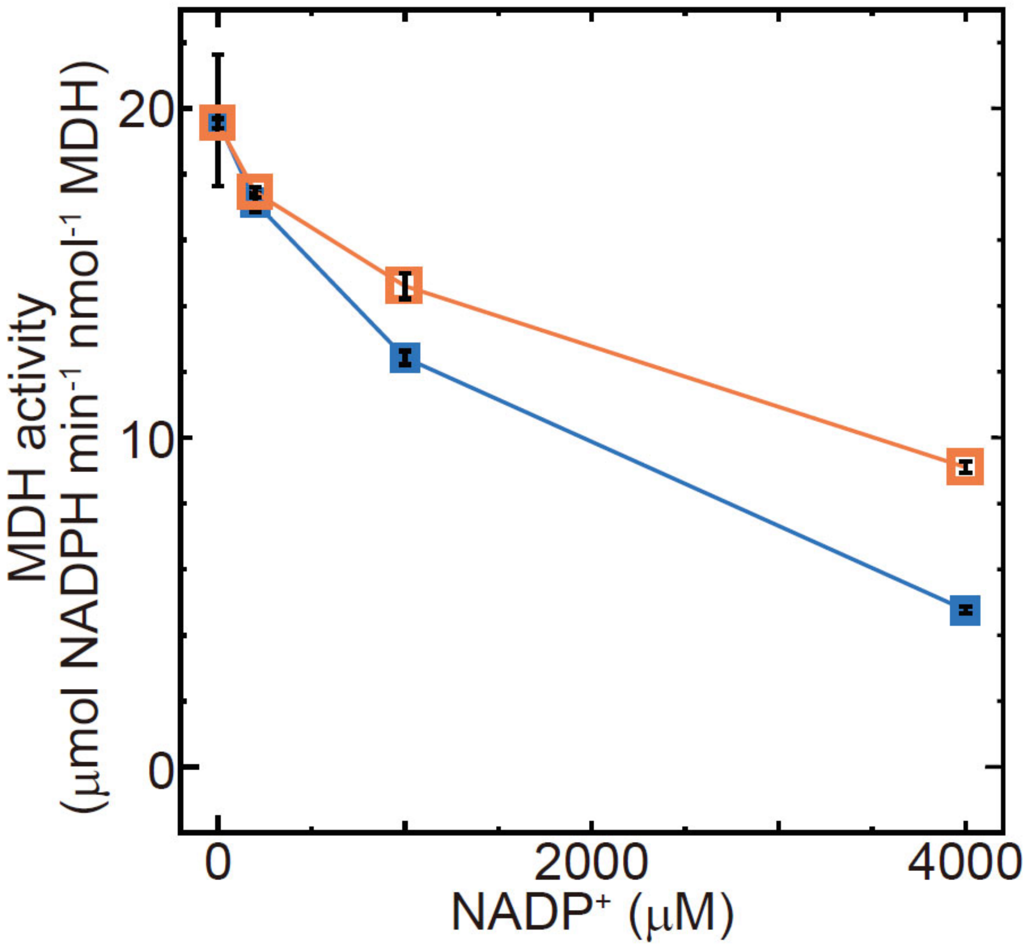

SI appendix Fig. S10. Feedback inhibition of the activities of MDH_{WT} (blue symbol) and $MDH_{\Delta C}$ (orange symbol) by $NADP^+$. Buffer containing 50 mM Tris-HCl (pH 7.5), 50 mM NaCl, 0.01 μM reduced MDH_{WT} or $MDH_{\Delta C}$, 100 μM NADPH, 200 μM oxaloacetate, and 0–4000 μM $NADP^+$ was used. Values are presented as mean ± SD (n = 3, one measurement for each independently prepared (pre-treated) sample). For the data of 0 μM $NADP^+$, the data of 200 μM oxaloacetate in Fig. 1D were used.

SI appendix References

1. Hahn F, Eisenhut M, Mantegazza O, Weber APM. Generation of Targeted Knockout Mutants in Arabidopsis thaliana Using CRISPR/Cas9. *Bio-Protocol* **7**, (2017).
2. Wakao S, Benning C. Genome-wide analysis of glucose-6-phosphate dehydrogenases in Arabidopsis. *Plant J* **41**, 243–256 (2005).
3. Yoshida K, Hisabori T. Two distinct redox cascades cooperatively regulate chloroplast functions and sustain plant viability. *Proc Natl Acad Sci U S A* **113**, E3967–3976 (2016).

SI appendix Table S1. Result of the metabolome analysis.

Metabolite	End of dark		End of light	
	Control-CR	$MDH_{\Delta C}$-CR	Control-CR	$MDH_{\Delta C}$-CR
Ornithine	2.37 ± 0.36	2.81 ± 0.62	3.33 ± 0.50	3.11 ± 0.43
Lys	16.0 ± 1.2	14.8 ± 0.89	11.3 ± 0.80	9.53 ± 1.3
Arg	7.85 ± 1.9	8.70 ± 1.0	16.5 ± 1.2	18.9 ± 1.6
Ala	454 ± 74	875 ± 290	574 ± 61	675 ± 54
Asn	316 ± 32	282 ± 15	314 ± 27	345 ± 18
Gln	1,210 ± 140	755 ± 45	2,190 ± 180	2,140 ± 120
Glu	2,830 ± 200	3,060 ± 170	2,040 ± 100	1,630 ± 160
Asp	1,590 ± 120	1,350 ± 200	1,840 ± 80	1,390 ± 110
His	17.7 ± 3.8	17.3 ± 1.4	28.3 ± 1.1	28.7 ± 2.8
Gly	26.0 ± 24	19.0 ± 5.5	252 ± 68	268 ± 89
Ile	10.4 ± 3.2	9.20 ± 1.0	15.4 ± 0.92	13.3 ± 1.1
Leu	10.5 ± 5.2	11.0 ± 1.9	10.6 ± 0.52	11.1 ± 1.7
Met	3.91 ± 0.61	3.42 ± 0.42	12.1 ± 1.6	9.01 ± 1.5
Pro	131 ± 17	197 ± 52	138 ± 30	173 ± 37
Phe	19.2 ± 3.1	15.6 ± 1.3	51.7 ± 3.8	40.3 ± 8.5
Cys	2.72 ± 0.66	2.39 ± 0.58	2.86 ± 0.84	3.77 ± 0.89
GABA	12.1 ± 4.9	54.8 ± 62	4.14 ± 1.1	7.54 ± 4.1
Ser	327 ± 130	271 ± 51	803 ± 110	995 ± 72
Val	35.3 ± 5.7	34.6 ± 1.9	48.5 ± 2.1	45.7 ± 3.7
HomoSer	1.11 ± 0.27	1.24 ± 0.21	7.78 ± 0.65	5.55 ± 1.0
Thr	367 ± 100	157 ± 27	850 ± 38	519 ± 75
Trp	6.02 ± 1.4	5.34 ± 1.1	8.04 ±1.0	6.71 ± 0.68
Citrulline	32.4 ± 3.4	19.1 ± 2.0	138 ± 12	122 ± 15
Tyr	3.46 ± 1.7	4.54 ± 1.2	8.31 ± 0.31	7.07 ± 1.3
Aconitate	109 ± 6.4	99.4 ± 9.2	26.2 ± 19	21.6 ± 7.4
Isocitrate	34.5 ± 12	35.6 ± 5.4	26.3 ± 4.5	36.0 ± 3.1
Citrate	21,600 ± 1,900	19,100 ± 1,700	3,950 ± 1,300	7,940 ± 1,200
Fumarate	4,610 ± 550	3,160 ± 520	8,530 ± 630	6,410 ± 210
Malate	4,690 ± 730	3,880 ± 850	7,020 ± 570	9,000 ± 490
2OG	22.8 ± 3.4	19.5 ± 4.2	12.2 ± 7.0	3.62 ± 3.5
Succinate	332 ± 35	253 ± 92	49.6 ± 25	24.1 ± 22

SI appendix Table S1. (Continued)

Metabolite	End of dark		End of light	
	Control-CR	$MDH_{\Delta C}$-CR	Control-CR	$MDH_{\Delta C}$-CR
Lactate	84.9 ± 35	115 ± 70	51.1 ± 24	47.2 ± 15
Ascorbate	1,360 ±130	1,330 ± 130	1,890 ± 180	1,480 ± 77
PEP	12.9 ± 1.2	8.19 ± 5.0	21.4 ± 4.9	26.9 ± 5.4
PGA	53.2 ± 6.7	37.8 ± 8.6	168 ± 31	187 ± 51
FBP	0.333 ± 0.31	N.D.	0.119 ± 0.27	0.310 ± 0.43
DHAP	0.486 ± 1.1	0.668 ± 1.5	9.13 ± 3.3	7.17 ± 2.3
G-3P	5.16 ± 1.9	4.72 ± 1.2	11.2 ± 3.0	10.7 ± 0.74
GAP	N.D.	N.D.	N.D.	2.37 ± 2.5
G1P	7.63 ± 1.6	6.16 ± 2.9	13.7 ± 4.9	17.5 ± 1.4
F6P	32.3 ± 3.9	27.0 ± 3.3	78.2 ± 6.8	69.4 ± 6.5
G6P	148 ± 13	137 ± 11	262 ± 23	209 ± 17
RuBP	0.415 ± 0.58	N.D.	4.07 ± 1.6	6.11 ± 2.8
6PG	0.151 ± 0.34	0.261 ± 0.58	N.D.	0.342 ± 0.77
Ru5P	0.379 ± 0.35	1.61 ± 0.99	3.52 ± 0.86	4.03 ± 1.5
R5P	0.0926 ± 0.21	1.17 ± 0.68	3.44 ± 0.62	2.74 ± 0.65
S7P	2.77 ± 0.51	43.9 ± 8.7	50.3 ± 6.2	32.9 ± 5.4
Cinnamate	41.3 ± 3.9	45.1 ± 5.9	29.7 ± 2.2	25.1 ± 3.7
Shikimate	26.4 ± 6.7	53.1 ± 5.7	38.3 ± 9.6	60.9 ± 16

Each value represents the mean (μmol/g fresh weight) ± SD (n = 5, biological replicates).
N.D.: not detected.

SI appendix Table S2. Primers used in this study.

Name	Sequence	Purpose
MDH mature F	ACATATGTCCGTTTCTCAAAATAGC	MDH plasmid construction
MDH mature R	GAAACGGACATATGTATATCTCCTTC	MDH plasmid construction
MDH ΔC F	GCCTACTAGTGATCTTGGTCCGGTAG	Site-directed mutagenesis
MDH ΔC R	AGATCACTAGTAGGCAATGCCCTCTC	Site-directed mutagenesis
MDH C430S F	GCCTACTCAGATCTTGGTCCGGTAGAT	Site-directed mutagenesis
MDH C430S R	AAGATCTGAGTAGGCAATGCCCTCTCC	Site-directed mutagenesis
MDH C418S F	AAGAGATCAGTTGCACACCTCACTGGA	Site-directed mutagenesis
MDH C418S R	TGCAACTGATCTCTTCTCAGCCAACAG	Site-directed mutagenesis
MDH C77S F	AAAGAGTCATATGGAGTGTTCTGCCTC	Site-directed mutagenesis
MDH C77S R	TCCATATGACTCTTTCTTCGTCTTCAC	Site-directed mutagenesis
MDH C82S F	GTGTTCTCACTCACCTATGATCTTAAA	Site-directed mutagenesis
MDH C82S R	GGTGAGTGAGAACACTCCATATGACTC	Site-directed mutagenesis
sgRNA1 F	ATTGAGATACGTACCTCTCCAGTG	sgRNA subcloning
sgRNA1 R	AAACCACTGGAGAGGTACGTATCT	sgRNA subcloning
sgRNA2 F	ATTGTACCGGACCAAGATCACAGT	sgRNA subcloning
sgRNA2 R	AAACACTGTGATCTTGGTCCGGTA	sgRNA subcloning
FH41[a]	AAACGACGGCCAGTGCCAGAATTGGG-CCCGACGTCG	CRISPR plasmid construction
FH42[a]	TACTGACTCGTCGGGTACCAAGCTAT-GCATCCAACGCG	CRISPR plasmid construction
FH254[a]	GCCCAATTCCAAGCTATGCATCCAAC-GCG	CRISPR plasmid construction
FH255[a]	CATAGCTTGGAATTGGGCCCGACGTCG	CRISPR plasmid construction
FH61[a]	CTGCCCAACGAGAAGGTGC	Screening
FH201[a]	AGTCGGACAGGCGGTTGATG	Screening
MDH gDNA F	GGTTAGAAGAGGGATTCACTGAG	Screening, cDNA sequencing
MDH gDNA R	ACTGGAATAAAGCATAGAAACG	Screening
MDH seq	TTCTGCTGCTTCTACTGCTG	gDNA sequencing
G6PDH1 F	GTTTTGCTACTAATTTAGACAATGCAAC	qPCR
G6PDH1 R	TATCTCTCTTGGATACCTGGATCG	qPCR
G6PDH2 F	CTTTATGCAAGATGGTGCAATAGTC	qPCR
G6PDH2 R	GGCTTTGACCATCAGATTCTACTC	qPCR

SI appendix Table S2. (continued)

Name	Sequence	Purpose
G6PDH3 F	CGCTGATCAATGTTGTTGACCC	qPCR
G6PDH3 R	TGGCTACTGATCCATCTTGCAC	qPCR
G6PDH4 F	TTGCTCTATAAAGACAGGTATAAAACCG	qPCR
G6PDH4 R	GCTGTGTGATGCTTGTCTATCTCTTC	qPCR
G6PDH5 F	TGTAAAAGAATACAATCCAGTCACG	qPCR
G6PDH5 R	GGATTAAGAAATCCCTGATGAAATAG	qPCR
G6PDH6 F	TGATTCGTTTGTGAGAGAATATGG	qPCR
G6PDH6 R	ATTGAGAAATCCCTGACGGTATAAG	qPCR
RPS15aA F[b]	GAAGCACGGTTACATTGGTG	qPCR
RPS15aA R[b]	TGTCTAGAAGGGAGCAAACG	qPCR
HygR F	GCGATGGACTAGTGCTTGCTCTAAAC-CTCGTTC	HygR cassette exchange
HygR R	GGAACTGACTAGTAGCAGCTTGCCAA-CATGG	HygR cassette exchange
pRI201 (cassette exchange) F	AGCTGCTACTAGTCAGTTCCAAACGT-AAAACGG	HygR cassette exchange
pRI201 (cassette exchange) R	AGCAAGCACTAGTCCATCGCCCTGAT-AGACGG	HygR cassette exchange
MDH transit F	TCACTGTTGATACATATGGCCATGGC-AGAGCTCTC	MDH-OE plasmid construction
MDH transit R	CTTGGCTATTTTGAGAAACGG	MDH-OE plasmid construction
MDH in pET23c R	AGAATTGTCGACAGCCGGATCTCAGTGG	MDH-OE plasmid construction
pRI201 (MDH integration) F	TCCGGCTGTCGACAATTCTGAATCAAC	MDH-OE plasmid construction
pRI201 (MDH integration) R	GCCATATGTATCAACAGTGAAG	MDH-OE plasmid construction
Salk LBb1.3[c]	ATTTTGCCGATTTCGGAAC	Screening

[a]Primers used in a previous study [1].

[b]Primers used in a previous study [3].

[c]Primer used for Salk line screening (http://signal.salk.edu/).
